# Enzyme-Linked Immunosorbent Assay and Serologic Responses to *Pneumocystis jiroveci*

**DOI:** 10.3201/eid1005.030497

**Published:** 2004-05

**Authors:** Kieran R. Daly, Judy Koch, Linda Levin, Peter D. Walzer

**Affiliations:** * University of Cincinnati College of Medicine, Cincinnati, Ohio, USA; ‡ Veterans Affairs Medical Center, Cincinnati, Ohio, USA

**Keywords:** Pneumocystis, Major surface glycoprotein, Infection, ELISA, HIV patients, Serum antibodies

## Abstract

Seroepidemiologic studies of *Pneumocystis* pneumonia (PCP) in humans have been limited by inadequate reagents. We have developed an enzyme-linked immunosorbent assay (ELISA) using three overlapping recombinant fragments of the human *Pneumocystis* major surface glycoprotein (MsgA, MsgB, and MsgC) for analysis of antibody responses in HIV-positive patients and healthy blood donors. HIV-positive patients had significantly higher antibody levels to all Msg fragments. Furthermore, HIV-positive patients who experienced a previous episode of PCP (PCP-positive) had higher level of antibodies to MsgC than patients who never had PCP. A significant association was found between ELISA antibody level and reactivity by Western blot in HIV-positive patients, especially those who were PCP-positive. Thus, this ELISA will be useful in studying serum antibody responses to *Pneumocystis* in different human populations.

*Pneumocystis* is an opportunistic fungus of worldwide distribution that can cause lethal pneumonia in persons infected with HIV and in other persons with depressed immune function (*1*). *Pneumocystis* also infects a variety of animals and causes pneumonia in those that are immunodeficient or immunosuppressed (*2*). Since *Pneumocystis* organisms in humans and animals are very similar, and the pneumonia that develops has many common features, these microbes were originally thought to belong to a single genus and species. However, data over the past decade have shown that *Pneumocystis* is genetically diverse and host specific, suggesting that studies of immune responses to *Pneumocystis* are best performed by using organisms or organism products that are specific for that host (*1*). *Pneumocystis* nomenclature, which is evolving and somewhat controversial, has designated two species so far: *P. carinii* for rat-derived organisms and *P. jiroveci* for human-derived organisms (*3*).

Seroepidemiologic surveys have shown that exposure to *Pneumocystis* occurs early in life; by 2 to 4 years of age, most children have antibodies to the organism (*4*–*8*). Since a reliable in vitro culture system for *Pneumocystis* has not been developed, antigens used in these studies consisted mainly of whole or fractionated organism preparations derived from infected human or rodent lungs. The use of human *Pneumocystis* has been further hindered by the small amounts of material that can be obtained from clinical specimens. Serologic surveys using crude *Pneumocystis* antigen preparations are generally not effective as clinical or epidemiologic tools. The frequency or level of serum antibodies to *Pneumocystis* among HIV-positive patients and other immunocompromised hosts has usually been similar to the corresponding value in healthy controls (*6*,*8*–*15*). Conflicting results have been obtained in attempts to distinguish past from present infection or colonization from active disease (*16*–*21*). Thus, no standardized antigen preparations are useful for antigen-specific immunologic studies of *Pneumocystis* infection in humans.

The *Pneumocystis* antigen that has received the most attention is the 95- to 140-kDa major surface glycoprotein (Msg or gpA), which contains shared and species-specific epitopes, elicits humoral and cellular protective immune responses, and plays a central role in the interaction of *Pneumocystis* with its host (*2*–*8*,*22*). However, native *P. jiroveci* Msg is in short supply and contains multiple isoforms of this glycoprotein, which complicates immunologic studies of this antigen (*23*,*24*).

Recombinant *Pneumocystis* antigens offer a viable approach to developing novel reagents for use in immunologic assays (*25*–*27*). One group of investigators has developed two recombinant fragments that correspond to the amino and carboxyl halves of Msg (*25*,*26*). In Western blot analysis, the carboxyl fragment was recognized more frequently by serum specimens than the amino fragment but did not distinguish between HIV+ patients and healthy donors. An enzyme-linked immunosorbent assay (ELISA) using the carboxyl fragment showed significantly higher antibody levels in HIV-negative patients with *Pneumocystis* pneumonia (PCP) compared with healthy controls, but it could not distinguish among HIVpositive, PCP-positive and HIV-positive, PCP-negative patients and controls.

We recently developed three overlapping recombinant fragments of Msg that span the length of *P. jiroveci* Msg and used these antigens to measure serum antibodies by Western blot in healthy blood donors and HIV patients (*27*). The data showed significant differences in the frequency of reactivity to the Msg constructs, not only between the two groups but also between PCP-positive and PCP-negative patients with HIV.

Although Western blot is a valuable serologic technique, its major limitation is that it is not quantitative. ELISA overcomes this problem and is better suited for population surveys. We developed an ELISA using recombinant Msg fragments as antigens and analyzed antibody responses of healthy blood donors and HIV-positive patients (including PCP-positive and PCP-negative patients). We present three different analyses of the ELISA data and compare the results to those obtained by Western blot.

## Materials and Methods

Serum samples as well as the demographic and clinical information about the human participants in this study were described in detail in our earlier report (*27*). That report also describes how recombinant Msg fragments were cloned, expressed, and purified and how Western blot was performed.

### Enzyme-linked Immunosorbent Assay

Serum specimens were tested against the following antigens: recombinant Msg fragments; *Escherichia coli* extract expressing the pET vector without insert as a vector control; tetanus toxoid (TT) as a positive control; phosphate-buffered saline (PBS) without antigen (negative control). A standard serum sample, obtained from a healthy donor with known reactivity to MsgA, MsgB, and MsgC in Western blot, is run on each plate as a control. Duplicate wells of a 96-well plate were coated with antigen (1 μg/mL, 100 μL/well in PBS pH 7.4 overnight. The plates were washed in wash buffer (PBS with 0.05% Tween-20) and blocked with blocking buffer (wash buffer with 5% nonfat milk) (200 μL/well) for 2 h at room temperature. The plates were washed again, and human serum diluted 1/100 in blocking buffer was added to each well (100 μL/well). The plates were rocked overnight at 4°C, washed in wash buffer, and horseradish peroxidase (HRP)-labeled goat anti-human immunoglobulin (Ig) G (heavy and light chains) was added to each well (100 μL/well at 1/5,000 dilution in blocking buffer). HRP-labeled S-protein was used on each plate as a positive control and to correct for antigen loading. The plates were incubated at room temperature for 1 h, washed, and developed by adding 3,3′,5,5′-tetramethylbenzidine substrate (100 μL/well). Color was allowed to develop for 4 min, the reaction was stopped by adding 100 μL of 0.18 mol/L H_2_SO_4_ to each well, and the plates were read at a wavelength of 450 nm.

Data were analyzed three different ways. In ELISA 1, the reactivity of each serum specimen to Msg was expressed as the ratio of reactivity to the pET vector (mean optical density [OD] Msg_test serum_ – mean OD PBS_test serum_) / (mean OD pET_test serum_^__^ mean OD PBS_test serum_). In ELISA 2, the reactivity to Msg was expressed as the percent reactivity to TT for each serum: (mean OD Msg_test serum_^__^ mean OD PBS_test serum_) / (mean OD TT_test serum_^__^ mean OD PBS_test serum_) x 100. In ELISA 3, the reactivity to Msg was expressed as percent reactivity of the standard serum: (mean OD Msg_test serum_ – the mean OD PBS_test serum_) / mean OD Msg_standard serum_ – the mean OD PBS_standard serum_) x 100. Variations in assay results using the control serum were measured for MsgA and MsgC on a per-plate basis (n = 6), a daily basis using two plates (n = 12), and an overall basis (across 4 days with two plates per day) (n = 48). The coefficients of variation for MsgA were 3%–5%, 3.3%–5.8%, and 8.7%, respectively, and for MsgC, 3.6%–7%, 4.8%–7.4%, and 13.3%, respectively.

### Statistics

Geometric means and 95% confidence intervals (95% CI) of observed ELISA measurements were obtained by patient category and antigen status. Before analysis, ELISA measurements were log-transformed to approximate normality. Weighted least squares regression analysis of variance (ANOVA) was carried out for each ELISA measurement separately to test the equality of ELISA means between PCP-positive, PCP-negative, HIV, and donor categories, adjusted for categorically modeled antigen level (A, B, C). The weighting values were the inverse of the within-category ELISA variances. The means of PCP-positive, PCP-negative, and HIV groups were compared to the mean of blood donors by calculating a post hoc linear contrast of individual estimates. The model included interactions between antigens and patient categories to allow for possible differences in the effect of patient status on mean values of ELISA among antigen levels. Significance was judged as p < 0.05, unless stated otherwise.

The association between Western blot positivity and ELISA was investigated by logistic regression in which Western blot (dichotomous ±) was related to continuously measured ELISA, adjusted for categorically modeled antigen level (A, B, C) and patient group (PCP-positive, PCP-negative, HIV, donor). Associations were obtained by calculating the odds of Western blot positive versus negative results for increasing ELISA reactivity, ranging from low (25th percentile) to high (75th percentile). From the same analysis, odds ratios (ORs) measuring associations between Western blot positivity and patient status (donor versus PCP-positive, PCP-negative, HIV) were calculated at midpoints of ELISA 1, 2, and 3, equal to 2.7, 16, and 35, respectively. ORs for the combined PCP groups, i.e., blood donor versus HIV, were obtained by comparing the mean of PCP groups to mean of the donor group, calculated from a linear contrast of the individual group effects. Significance was judged by obtaining 95% CI of ORs. A CI that does not include 1 indicates a significant association (two-sided p = 0.05) between the odds of Western blot reactivity when ELISA results are high versus low, or between blood donors and patient groups at the midpoint of ELISA measurements.

Spearman correlation coefficients were obtained by measuring associations between ELISA and CD4 and viral counts in HIV patients. All statistical analyses were performed by using the SAS statistical analysis system (SAS for Windows, Version 8.2, SAS Institute, Cary, NC.)

## Results

### Analysis of HIV-Positive and Blood Donor Serum Specimens

The reactivity of serum antibodies from HIV patients and healthy blood donors to MsgA, MsgB, and MsgC was compared by three different ELISA analyses (Table 1). Irrespective of the method of analysis, HIV-positive patients had significantly higher levels of antibody than healthy blood donors to each of the Msg fragments (p < 0.001), even though the range of ELISA values overlapped between the two groups (Figure). In blood donors, antigen reactivity was hierarchical (MsgC > MsgA > MsgB) for all methods of analysis. Similar results were seen for HIV patients in ELISA 1 and 2, but in ELISA 3 reactivity to MsgB was higher than to MsgA or MsgC (not significant).

**Table 1:** Geometric mean values and 95% CI of ELISA by blood donor, PCP-positive, PCP-negative, and HIV status for each antigen^a^

ELISA	Antigen	Blood donor, n = 95 (95% CI)	PCP+, n = 33 (95% CI)	PCP–, n = 66 (95% CI)	HIV+ (PCP+, PCP–), n = 94 (95% CI)
1 (n = 189)	A	1.2 (0.8 to 1.8)	6.8 (4.1 to 11.3)	6.0 (4.6 to 7.7)	6.2 (4.9 to 7.9)
	B	1.0 (0.7 to 1.4)	3.7 (2.4 to 5.8)	2.9 (2.3 to 3.6)	3.2 (2.5 to 3.9)
	C	2.0 (1.2 to 3.1)	13.0 (7.9 to 21.4)	7.5 (6.0 to 9.3)	9.1 (7.3 to 11.4)
2 (n = 189)	A	9.0 (6.3 to 12.7)	31.2 (22.0 to 44.2)	28.9 (21.2 to 39.5)	29.7 (23.6 to 37.4)
	B	4.2 (2.7 to 6.6)	16.2 (11.5 to 22.7)	15.0 (11.9 to 18.9)	15.4 (12.8 to 18.6)
	C	21.4 (15.0 to 30.7)	57.8 (48.1 to 69.3)	39.1 (31.7 to 48.2)	44.9 (38.5 to 52.2)
3 (n = 189)	A	18.6 (10.5 to 32.9)	73.5 (53.0 to 102.1)	73.8 (54.1 to 100.7)	73.7 (58.6 to 92.7)
	B	8.2 (4.0 to 16.6)	102.6 (73.0 to 144.2)	103.8 (83.0 to 129.7)	103.4 (86.1 to 124.2)
	C	22.1 (12.6 to 38.6)	120.1 (99.8 to 144.5)	81.2 (66.7 to 98.9)	93.2 (80.5 to 107.9)

^a^CI, confidence interval; ELISA, enzyme-linked immunosorbent assay; PCP, *Pneumocystis* pneumonia.

**Figure:**
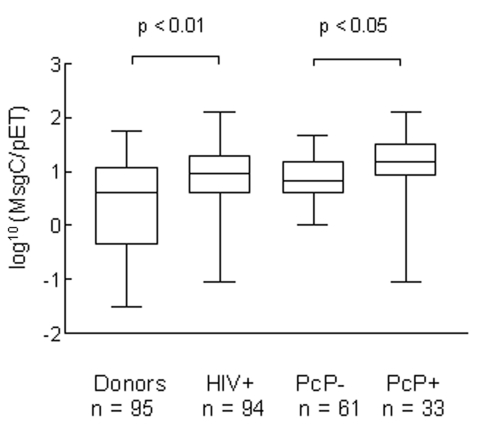
Antibody reactivity of healthy blood donors (donors); HIV-positive; PCP-positive, HIV-positive; and PCP-negative, HIV-positive patients to human *Pneumocystis* major surface glycoprotein C (MsgC) by enzyme-linked immunosorbent assay 1 (ratio to pET), showing the range, 25% and 75% confidence intervals, and median of the data. Data were log-transformed to approximate normality.

To determine if the increased reactivity of the HIV serum samples could be accounted for by increased antibodies in the PCP-positive patients as a consequence of infection with *Pneumocystis*, we compared the reactivity of HIV-positive, PCP-negative and HIV-positive, PCP-positive patients with that of blood donors (Table 1, Figure). Irrespective of the ELISA method chosen, the reactivity of PCP-negative serum samples was higher than that of blood donor serum samples for each of the Msg fragments (p < 0.01), indicating that PCP status alone could not account for the increased antibody reactivity found in the HIV cohort.

### Comparison of PCP-Negative and PCP-Negative Serum Specimens

PCP-positive patients had significantly higher reactivity to MsgC than PCP-negative patients by all three methods of analysis (p < 0.05, p < 0.01, and p < 0.01 for ELISA 1, 2, and 3, respectively). Reactivity to MsgA or MsgB did not significantly differ between the populations by any method of analysis.

In ELISA 1, MsgC elicited the highest reactivity in both PCP-positive and PCP-negative groups, which was significant when compared to reactivity to MsgB (p < 0.001 in both groups) but not to MsgA (p nonsignificant in both groups). In ELISA 2, the recognition of MsgC by PCP-positive patients was significantly higher than that of MsgA or MsgB, and MsgA exhibited higher reactivity than MsgB (p < 0.01, all comparisons). Similarly, in PCP-negative patients, the highest reactivity in ELISA 3 was to MsgC, which was significant when compared to MsgB (p < 0.01) but not compared to MsgA (p = 0.11). In contrast to the other ELISA methods, the highest levels within each group were to MsgB, but this finding was not significant (p > 0.05). However, level of reactivity to MsgC was significantly higher than to MsgA in the PCP-positive cohort (p < 0.01).

### Correlation between ELISA and Western Blot

To determine whether a correlation existed between ELISA values and reactivity by Western blot analysis, we compared data obtained by ELISA with Western blot results by logistic regression. As demonstrated by the adjusted ORs and 95% CI, a significant association was found between seropositivity by Western blot and higher ELISA values among HIV-positive patients (p < 0.05) but not among blood donors (Table 2). This association was found for reactivity to all Msg fragments and all ELISA methods, with the highest association being found for ELISA 3. Significant and positive associations were also found between Western blot reactivity and blood donor/HIV status at ELISA midpoints (geometric mean values shown by ELISA) (Table 3). Again, the highest value was found with ELISA 3.

**Table 2:** Adjusted odds ratios (95% CI) measuring the associations between Western blot reactivity and ELISA values (25–75 percentile increase) among 189 study participants^a,b^

ELISA	Blood donor, n = 95 (95% CI)	PCP+, n = 33 (95% CI)	PCP–, n = 61 (95% CI)	HIV, n = 94 (95% CI)
1	1.1 (0.9 to 1.4)	5.1 (2.2 to 11.8)	1.4 (0.8 to 2.6)	2.7 (1.2 to 5.8)
2	1.1 (0.9 to 1.4)	20.4 (6.4 to 65.1)	3.6 (2.0 to 6.6)	8.6 (4.4 to 16.9)
3	1.0 (0.9 to 1.1)	11.6 (3.6 to 37.9)	7.4 (3.6 to 15.1)	9.3 (4.7 to 18.5)

^a^CI, confidence interval; ELISA, enzyme-linked immunosorbent assay; PCP, *Pneumocystis* pneumonia. ^b^Odds ratios were obtained by using logistic regression. Positive associations were found between Western blot reactivity and increasing ELISA. Based on 95% confidence intervals, the odds of Western blot reactivity were significantly related (p < 0.05) to increasing ELISA 1, 2, and 3 among PCP+ patients and HIV patients across all antigens. For PCP– patients this result was true for ELISA 2 and 3 only. No significant results were found for the blood donor group.

**Table 3:** Adjusted odds ratios (95% CI) measuring the associations between Western blot reactivity and participant status (blood donor versus PCP-positive, PCP-negative, HIV) among 189 study participants^a,b^

ELISA	Midpoint	Blood donor: PCP+ (95% CI)	Blood donor: PCP– (95% CI)	Blood donor: HIV (95% CI)
1	2.7	3.2 (1.6 to 6.7)	2.2 (1.4 to 3.5)	2.7 (1.7 to 4.3)
2	16	5.9 (2.5 to 14.3)	3.2 (2.0 to 5.3)	4.3 (2.6 to 7.4)
3	35	10.0 (2.9 to 33.3)	9.1 (4.4 to 20.0)	9.4 (4.6 to 19.3)

^a^CI, confidence interval; ELISA, enzyme-linked immunosorbent assay; PCP, *Pneumocystis* pneumonia. ^b^Odds ratios measuring the association between Western blot reactivity and participant status were calculated at the midpoints of ELISA measurements over all antigens. Based on 95% confidence intervals, blood donors had significantly increased odds of Western blot reactivity compared to each patient group (p < 0.05) for each ELISA.

Tables 2 and 3 show results of logistic regression analyses relating ELISA values and Western blot reactivity in PCP-positive and PCP-negative patients. A significant association was found between increasing ELISA 1 values and Western blot seropositivity in PCP-positive patients only (p < 0.05). Similar results were found for each Msg fragment in each patient group. Also, a significant association between increasing ELISA 2 levels and Western blot positivity in PCP-positive patients was found (p < 0.05). The relationship between ELISA 2 measurements and Western blot reactivity varied by Msg fragment in PCP-negative patients; a significant association was found for Msg C only (p < 0.05, OR = 16.9, 95% CI = 3.6 to 79.7). For ELISA 3, a significant OR was found for both patient groups (p < 0.05). Significant and positive associations were also found between Western blot reactivity and blood PCP status at ELISA midpoints (geometric mean values of ELISA) (Table 3), with the highest value found for ELISA 3. Spearman correlations were obtained between ELISA values and CD4+ cell counts or viral titer in PCP-positive and PCP-negative patients. No significant associations were noted (data not shown).

In our earlier study, PCP developed in 9 of the 61 PCP-negative patients after their serum samples had been obtained. None of these persons showed antibodies to MsgC by Western blot. These patients had a geometric mean antibody level in ELISA 1 o f 5.47 (95% CI 2.57 to 11.64) compared with a level of 7.88 (95% CI 6.25 to 9.94) in the 52 PCP-negative patients. Although mean serum levels were not significantly different among patient groups (p > 0.05), some trends were interesting. Patients in whom PCP developed subsequent to collecting their specimens had lower antibody levels to MsgC than the other patient groups.

## Discussion

Using ELISA, we have compared blood donor and HIV+ patient serum samples for antibody reactivity to recombinant fragments of *P. jiroveci* Msg. We analyzed the data by three independent methods and found that HIV-positive patients had significantly higher mean serum antibody levels to MsgA, MsgB, and MsgC than healthy blood donors by all methods of analysis. Furthermore, when the HIV-positive patients were separated on the basis of a prior documented episode of PCP, the PCP-positive patients had higher antibody levels to MsgC than the PCP-negative patients by all ELISA methods tested. These results differ from published work (*25*,*26*) showing no difference in antibody levels between HIV-positive, PCP-positive patients; HIV-positive, PCP-negative patients; and healthy controls to recombinant fragments of Msg. The discrepancy in results could be due to differences in study populations, Msg preparations, and methods (*28*).

At the onset of the study, we decided on three independent methods of analysis of the data in an attempt to standardize the assay. Each method of analysis provides different information about the results of the ELISA, and each method has strengths and weaknesses. The first analysis involved comparing the response to recombinant Msg fragments with the response to protein expressed from the pET vector without insert. The protein expressed from the sequences of the pET vector is an integral part of each Msg recombinant tested, and therefore this method of analysis allows measurement of Msg-specific immune reactivity corrected internally for any reactivity to the vector-derived sequences. The second method of analysis was a comparison of the reactivity to Msg with that of a standard antigen, tetanus toxoid, chosen because it is likely that most adults have been vaccinated against tetanus toxoid and are likely to react with this protein in assay. While this method is consistent when analyzing individual serum samples, one potential problem with this analysis is that the antibody response to tetanus toxoid varies from person to person. This variation means that, when a population is studied, the range of values obtained by this method can be quite large. The third method of analysis was comparing the test serum reactivity to that of a standard serum. Our standard serum was chosen because of clear reactivity to all Msg fragments tested in Western blot analysis. However, this serum responded weakly to MsgB in ELISA. Our choice of standard serum is still valid and is useful for comparing the reactivity to any one of the fragments in different populations. However, the use of this method to compare responses to different Msg fragments must be treated with caution. An alternative approach would be to use a pool of serum samples from several donors chosen for reactivity to the individual Msg fragments.

We have previously shown that MsgB was the most common fragment recognized by blood donors and HIV patients (*27*), and a significant difference was found in the frequency of recognition of MsgB between donors and HIV+ patients. In contrast to these results, this study has shown that MsgC is the antigen with the highest level of reactivity in both the healthy and HIV-positive populations tested. This apparent discrepancy between assays is probably due to the nature of the epitopes recognized in the different assays, since the recombinant antigens were reduced and denatured during testing by Western blot analysis but not in ELISA. The lack of strict concordance in recognition of Msg fragments in ELISA and Western blot has also been seen by other groups (*15*,*26*) However, we did find a significant association between ELISA antibodies and Western blot seropositivity in HIV-positive patients but not in blood donors. Using regression analysis, we calculated ORs measuring the association of Western blot positivity and study participant (blood donor vs. HIV) status at the midpoint of each ELISA. These results showed a significantly greater likelihood of being Western blot–positive among blood donors than HIV patients. These data can be explained by several factors. First, 84% of our blood donors, but only 66% of the HIV patients (p = 0.003), responded to at least one of the three Msg fragments. Second, blood donors have consistently lower ELISA values than HIV patients. Third, blood donors exhibit larger standard deviations than HIV patients in their ELISA values, suggesting they have a more heterogeneous antibody response in terms of affinity for Msg epitopes. The association between Western blot positivity and ELISA values is probably not due to the PCP-positive patients’ response, as PCP-negative patients also showed an association in two of the three ELISA methods.

Despite their lower Western blot seropositivity rate, HIV patients had significantly higher mean serum antibody levels to MsgA, MsgB, and MsgC than healthy blood donors by both methods of ELISA analysis. That HIV-positive patients should have higher levels of antigen-specific reactivity than healthy blood donors is surprising. One possible explanation is that HIV-positive patients may come into contact with *Pneumocystis* more frequently or for longer periods of time before the organism is cleared from the lungs (*1*). While this exposure to *Pneumocystis* may not result in overt PCP, the immune system likely takes longer to clear the organisms from the lung, allowing responses to new or subdominant epitopes to develop. In contrast, these responses would be absent or diminished in healthy populations, who would clear *Pneumocystis* from the lungs more quickly. Therefore, the higher response in HIV-positive patients could be due to the accumulated responses to multiple epitopes, whereas the responses in healthy blood donors may be limited to immunodominant epitopes. An alternative explanation may be that the elevated levels of reactivity seen in HIV-positive serum are specific for the IgG isotypes and that the elevated levels of the isotype profile of Msg-specific antibodies may be skewed in healthy persons and HIV-positive patients.

This study shows that PCP-positive patients have higher antibodies to MsgC than PCP-negative patients by all ELISA methods. Furthermore, nine HIV patients in whom PCP developed after their serum specimens had been obtained and who did not have antibodies to MsgC by Western blot (*27*) had lower levels of antibodies to MsgC in this study. Overall, a hierarchy appeared to exist in the level of serum antibodies to MsgC by ELISA: highest in PCP-positive patients, next highest in PCP-negative patients, and lowest in blood donors.

Humoral immunity has long been thought to have little role in host defenses against *Pneumocystis* because PCP develops in many patients despite preexisting antibodies to the organism (*8*). Yet considerable evidence, mainly from animal models, now suggests that B cells contribute to these host defenses (*2*). Also of interest is that some serologic studies have shown decreased antibody levels or production before a person acquires PCP or increased antibody levels after a patient recovers from PCP (*6*,*8*,*23*,*29*–*34*). Our data suggest that analysis of the immune reactivity to Msg fragments, MsgC in particular, may be important in understanding differences between patient populations and may lead to identifying epitopes linked to protection from or susceptibility to PCP in populations at risk.
